# Comment On: Encoding Genes of Metallo‑β‑Lactamases (IMP, NDM, and VIM) in *Klebsiella pneumoniae* in Iran: A Systematic Review and Meta‑Analysis (Sadeghi et al., 2025)

**DOI:** 10.1002/hsr2.71341

**Published:** 2025-10-09

**Authors:** Nandni Kumari, Joti Kumari, Muhammad Saad Khan

**Affiliations:** ^1^ Department of MBBS Liaquat University of Medical and Health Sciences Jamshoro Pakistan; ^2^ Department of MBBS Jinnah Sindh Medical University Karachi Pakistan

To the Editor,

We read with great interest the recent systematic review and meta‐analysis by Sadeghi et al., which estimates the prevalence of imipenemase (IMP), New Delhi metallo‐β‐lactamase (NDM), and verona integron‐encoded metallo‐β‐lactamase (VIM) genes in *Klebsiella pneumoniae* isolates across Iran from 2010 to 2024 [1]. As antimicrobial resistance (AMR) escalates globally, understanding the regional spread of resistance genes is essential for informing treatment, surveillance, and stewardship [2]. The authors commendably highlight limitations such as regional sampling gaps, language restrictions, and variability in diagnostic methods. However, several additional issues deserve consideration to enhance the interpretation and clinical utility of their findings.

First, the high heterogeneity reported (*I*² > 70%) across all three genes was not explored through subgroup, sensitivity, or meta‐regression analyses. Potential sources of this variability such as differences in detection techniques, patient populations, and geographic coverage remain unaddressed. Moreover, the long time span of the included studies raises the possibility of temporal bias. Without accounting for accumulation of studies reporting higher prevalence in recent years, the pooled estimates may not reflect true trends [3].

The study omits a trend analysis of gene prevalence over time. With global concern over rising NDM‐type carbapenemases, evaluating changes across the 14‐year period could have revealed important epidemiological shifts and guided resource allocation [3].

While methodological inconsistency among the included studies is acknowledged, the authors do not stratify results based on the diagnostic protocols used. Differences in PCR techniques or phenotypic confirmation likely impact sensitivity and comparability across sites [4]. Equally concerning is the absence of genotype–phenotype correlation. Knowing that a gene is present offers limited value if it is not linked to actual resistance phenotypes or treatment outcomes. Including data on minimum inhibitory concentration or clinical failure rates would have significantly strengthened the practical relevance of the review [4].

Finally, while the authors note publication and language bias, the exclusion of grey literature and hospital datasets may further skew regional representation and overestimate gene prevalence. This exclusion risks marginalizing data from under‐resourced settings, ironically where CRKP often poses the greatest clinical threat [4]. Moreover, failing to include nonindexed or non‐English studies may exacerbate funnel plot asymmetry and publication bias, as outlined by Egger et al. [5]. Integrating molecular prevalence data with clinical outcomes and antibiotic usage patterns would better inform antimicrobial stewardship protocols, particularly in resource‐limited Iranian settings.

In conclusion, the study provides important insight into the molecular epidemiology of metallo‐β‐lactamase‐encoding genes in Iran, but future research should incorporate analytical stratification, temporal modeling, and clinically connected data. Only through such enhancements can molecular surveillance data be transformed into actionable strategies that support national AMR control.

## Author Contributions

**Nandni Kumari:** conceptualization, methodology, writing – original draft. **Joti Kumari:** data curation, writing – original draft. **Muhammad Saad Khan:** writing – review and editing.

## Conflicts of Interest

The authors declare no conflicts of interest.

## Transparency Statement

The corresponding author affirms that this manuscript is an honest, accurate, and transparent account of the study being reported; that no important aspects of the study have been omitted.

## Data Availability

Data sharing is not applicable to this article as no datasets were generated or analysed during the current study.
